# Subacute left ventricle free wall rupture after acute myocardial infarction: awareness of the clinical signs and early use of echocardiography may be life-saving

**DOI:** 10.1186/1476-7120-4-46

**Published:** 2006-11-22

**Authors:** Luís Raposo, Maria João Andrade, Jorge Ferreira, Carlos Aguiar, Rute Couto, Miguel Abecasis, Manuel Canada, Nuno Jalles-Tavares, José Aniceto da Silva

**Affiliations:** 1 Cardiology Department, Hospital de Santa Cruz, Avenida Prof. Reinaldo dos Santos, 2790-134 Carnaxide, Portugal; 2 Cardiothoracic Surgery Department, Hospital de Santa Cruz, Avenida Prof. Reinaldo dos Santos, 2790-134 Carnaxide, Portugal; 3 Ressonância Magnética – Caselas – Bairro de Caselas, Rua Carolina Ângelo, 1400-045 Lisbon, Portugal

## Abstract

Left ventricular free wall rupture (LVFWR) is a fearful complication of acute myocardial infarction in which a swift diagnosis and emergency surgery can be crucial for successful treatment. Because a significant number of cases occur subacutely, clinicians should be aware of the risk factors, clinical features and diagnostic criteria of this complication. We report the case of a 69 year-old man in whom a subacute left ventricular free wall rupture (LVFWR) was diagnosed 7 days after an inferior myocardial infarction with late reperfusion therapy.  An asymptomatic 3 to 5 mm saddle-shaped ST-segment elevation in anterior and lateral leads, detected on a routine ECG, led to an urgent bedside echocardiogram which showed basal inferior-wall akinesis, a small echodense pericardial effusion and a canalicular tract from endo to pericardium, along the interface between the necrotic and normal contracting myocardium, trough which power-Doppler examination suggested blood crossing the myocardial wall. A cardiac MRI further reinforced the possibility of contained LVFWR and a surgical procedure was undertaken, confirming the diagnosis and allowing the successful repair of the myocardial tear. This case illustrates that subacute LVFWR provides an opportunity for intervention. Recognition of the diversity of presentation and prompt use of echocardiography may be life-saving.

## Background

Left ventricular free wall rupture (LVFWR) is a dramatic complication of acute myocardial infarction (AMI) and is presumably responsible for as much as 20 to 30% of all infarct related deaths [1-4]. Given the widespread use of reperfusion therapy, particularly mechanical reperfusion, the rate of occurrence as well as its classical clinical characteristics may have changed in the last one or two decades. However, the relatively consistent decrease in the autopsy rates probably renders its incidence under-reported [4]. Even considering that the occurrence of LVFWR may be substantially lower when a primary PCI can be performed [3], its incidence has been estimated to range from 0.7 to 8% and still, although rare, it appears to be 8 to 10 times more frequent than other fearsome mechanical complications of MI such as papillary muscle or interventricular septum rupture [3,5].

Historically, the first clinical reference to post-infarction left ventricular wall rupture was reported by William Harvey in 1647 [6], but it wasn't until 1972 that Fitz Gibbon and Montegut conducted the first successful operation for the correction of LVFWR due to ischemic heart disease [7,8].

We describe the case of a 69 year-old patient with sub-acute "contained" LVFWR which was successfully treated through surgical correction. The diagnosis was preliminarily established by bedside transthoracic echocardiography, driven by an unexpected change on a routine ECG.

## Case presentation

The patient was a 69 year-old caucasian male with recently diagnosed diabetes and a history of non-disabling stroke several years before. There was no record of hypertension, hypercholesterolemia, tobacco use or previously known coronary artery disease. Importantly, he was not taking any cardiovascular medication, including ASA.

The patient first presented to the emergency department of a referral hospital complaining of lower chest pain and vomiting. The ECG showed no signs of acute ischemia and, as biomarkers of myocardial necrosis tested negative, the patient was discharged with no further evaluation. During the next days, chest pain persisted and, on day five after the initial symptoms, he went back to the emergency. At that time the ECG showed inferior pathological Q waves, slight (~1 mm) ST-segment elevation and biphasic T waves on the inferior leads and a test for cardiac troponin I was positive. The patient was admitted with the diagnosis of subacute inferior myocardial infarction with recurrent angina and anti-thrombotic and anti-ischemic medications (including low molecular weight heparin and GP IIb/IIIa inhibitors) were started. Due to the persistence of chest pain the patient was referred for urgent coronary catheterization, which was performed at our institution on day five after symptom onset. Angiography showed a critical stenosis of the right coronary artery with TIMI 2 grade flow, with no other significant coronary lesions. An angioplasty procedure with deployment of a drug-eluting stent was successfully undertaken and the patient transferred to the CCU for routine monitoring.

**Figure 1:**
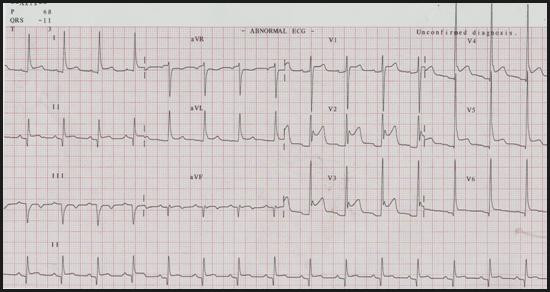
Surface 12-lead ECG on day 7 after the first episode of chest pain showing inferior Q-waves, "saddle-shaped" ST segment elevation and PR segment depression.

**Figure 2:**
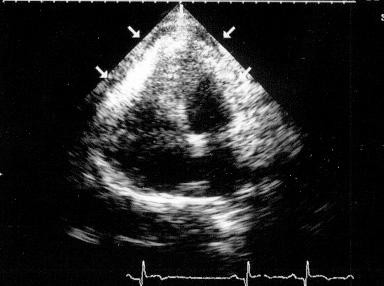
2D-Echo: apical four-chamber view showing a small global echodense pericardial effusion.

**Figure 3:**
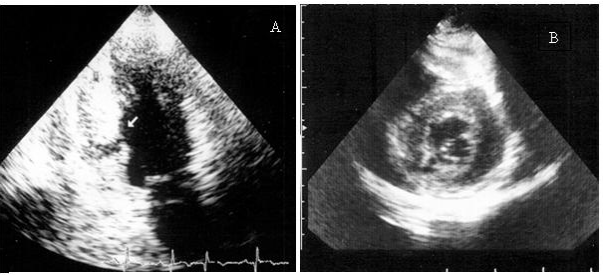
2D-Echo: apical two-chamber (A) and parasternal short axis (B) views showing a myocardial tear in the left ventricular inferior wall.

**Figure 4:**
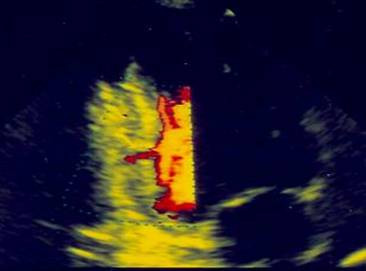
2D-Echo: apical two-chamber view with Power-Doppler suggesting blood flow across the inferior myocardial wall.

**Figure 5:**
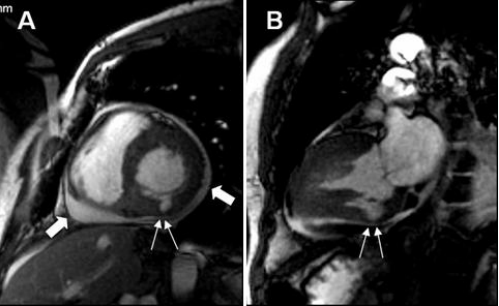
Cardiac MRI in short (A) and long (B) axis views showing pericardial effusion (large arrows) and thinning and dissection of the left ventricular inferior wall (small arrows).

On day seven after the first chest pain episode, clinicians were surprised by a new and persistent 4 to 5 mm ST-segment elevation affecting leads V1 to V5, I and aVL (Figure 1). The patient's clinical status remained remarkably stable as he experienced no recurrent chest pain, heart sounds could be eared clearly, there were no murmurs or bruits and blood pressure was in a somewhat expected range (~120/80 mmHg), considering that pressure lowering drugs had been newly introduced in incremental dosage. Serial evaluation of biomarkers of myocardial necrosis showed no increase relative to earlier post-PCI values, but white cell counts and CRP levels were both slightly elevated. At this point, a bedside transthoracic echocardiogram (TTE) was performed, showing a global small-to-moderate echodense pericardial effusion, somewhat larger in the posterior and inferior aspects of the heart, with no signs of tamponade (Figure 2 and Additional file 1). There were no regional wall motion abnormalities of the anterior wall and, in addition to basal inferior-wall akinesis, a canalicular tract from endo to pericardium was seen along the interface between the necrotic and the normal contracting myocardium (Figure 3, Additional file 2, Additional file 3, Additional file 4). Power-Doppler evaluation additionally suggested an abnormal blood leak across the inferior LV wall (Figure 4, Additional file 5) and, on the basis of these findings, a LVFWR was suspected. While the transoesophageal echocardiogram with intravenous echocontrast brought no further input, cardiac MRI images (Figure 5) corroborated the TTE findings and reinforced the diagnostic suspicion of LVFWR, leading to urgent surgery. After pericardiotomy and cloth evacuation there was a significant increase in the systemic blood pressure, suggesting that tamponade might have been impending. The myocardial tear was identified and successfully repaired with a tephlon band supported suture technique. Recovery was uneventful allowing hospital discharge 5 days after the operation. Two years after the event the patient is still alive and leading a normal life.

As previously mentioned, mechanical complications of acute MI, including LVFWR, apparently tend to become less frequent in day-to-day practice, at least in part due to the our growing ability to deliver safe and effective reperfusion therapies (both pharmacological and mechanical) to a wide range of MI patients.

### Timing of occurrence and risk factors for left ventricle wall rupture

The time frame for the occurrence of LVFWR may vary widely. In the pre-thrombolytic era incidence peaked between days 5 and 7 after the MI, but rupture has been reported to occur as late as one month or even beyond [9-11].

Traditionally, factors considered to increase the risk of LVFWR would include advanced age (6^th ^decade or later), female gender, hypertension without left ventricular hypertrophy, delayed or no reperfusion, the anterior location of the AMI, no prior angina, a first myocardial infarction, poor collateral circulation to the infarct related area, use of NSAIDs or steroids during the acute phase, and high serum levels of C-reactive protein [3,4,10].

Although cardiac rupture following AMI cannot be explained by any single factor, in this particular case, variables predicting the anticipated risk of cardiac rupture were related mainly to treatment delay and patient's age. Gender differences regarding the outcome after AMI have been well established, and even in the direct PCI (d-PCI) era, female sex has still been reported to be an independent predictor of LV rupture [3,12].

Despite being theoretically more probable in the inferior-lateral wall, it seems that the fact that anterior infarcts are more common, renders anterior wall ruptures more frequent [13]. However, a thorough literature review showed that, in the specific case of subacute LV wall rupture, inferior infarctions seem to be involved in the majority of the cases [14-16]. It has been stated that this might be due to the fact that patients with anterior wall ruptures may be less protected because blood tends to accumulate and to form adherent cloths in the posterior-inferior wall [17].

Despite age and general clinical status would have made this patient eligible for on site reperfusion therapy, absence or delay of timely reperfusion (owing to late diagnosis) was perhaps the major risk factor for LVFWR. The reported decrease in the incidence of MI-related cardiac rupture in the thrombolytic and subsequently in the d-PCI era is relatively consistent. Regarding the absolute risk of mechanical complications, there is general agreement in that *any reperfusion *is better than *no reperfusion*. However, perhaps particularly in elderly patients, the relation between the use and the timing of thrombolytic treatment (which is the most widely used reperfusion strategy) and the risk of LV rupture, has been matter of concern [1,18]. This is due to the fact that up to 30% of MI patients arrive to the hospital later than the optimal time-frame for thrombolysis to be potentially beneficial [5]. Although still controversial, there is evidence to suggest that, contrary to data provided by previous reports and *post-hoc *analysis of pivotal clinical trials [19,20], neither thrombolysis itself nor the time delay to thrombolytic treatment actually seem to increase the absolute risk of LVFWR. In the LATE trial [5], Becker *et al*. found no significant differences in the incidence of LVFWR in patients treated with a rt-PA based regimen within the 6 to 12 hour period after symptom onset and those randomized to the active treatment arm between 12 and 24 hours. However, there was a significant interaction between the assignment to active treatment and the time of rupture occurrence, as thrombolysis appeared to accelerate rupture events, typically to within 24 h after treatment [21]. Several possible mechanisms underlying this paradoxical effect of fibrinolysis have been proposed, such as extension of myocardial hemorrhage, weakening and dissection of the necrotizing zone [5], diminishing of the myocardial collagen content [22], and digestion of collagen by collagenases and plasmin [23-25]. Whether or not d-PCI would have similar effects has been recently examined in a retrospective analysis of a single centre registry of 1250 patients treated with d-PCI. In the 12 patients who had LV rupture (including free wall rupture and VSD), this complication occurred somewhat later than with thrombolysis, but earlier than that reported for non-reperfused patients [3]. The relative advantages of PCI versus lytics in this high risk group of patients have been evaluated in the SENIOR-PAMI trial [26]. In this yet unpublished study, 483 patients aged ≥ 70 years old were randomized to primary PCI or thrombolysis; despite a small difference favoring PCI, it failed to significantly reduce the 30-day mortality rate (10% vs 13%), thus suggesting that the incidence of fatal mechanical complications could have been quite similar. However, as the overall reduction in the incidence of the composite endpoint of death, MI or stroke favored PCI over thrombolysis (11.6 vs 18%; *p *= 0.05), this study might have been underpowered to evaluated the benefits of PCI in isolated hard endpoints such as MI-related death.

### Clinical presentation and diagnosis

When the acute form of LVFWR occurs, it usually results in an abrupt haemodynamic collapse with cardiac tamponade and electromechanical dissociation. Death ensues in a matter of minutes to hours in the large majority of cases, as cardiopulmonary resuscitation maneuvers are uniformly unsuccessful.

Less frequently, in up to one third of the cases, the rupture can be sealed by the epicardium or by a haematoma on the epicardial surface of the heart, forming a "LV diverticulum" or contained myocardial rupture. This situation represents a subacute pathologic condition standing somewhere between free rupture into the pericardial cavity and formation of a pseudoaneurysm [16,27-29]. This much less spectacular scenario – gradual or "oozing type" rupture – may evolve over hours or even days, and presents mainly with pericardial effusion related signs and symptoms.

Myocardial free wall rupture should be suspected in patients with recent MI who have recurrent or persistent chest pain, sometimes of the pericardial type, haemodynamic instability, syncope (resulting from transient electromechanical dissociation), and pericardial tamponade. New ST segment changes ("saddle shaped ST-segment elevation") may be the chief clinical manifestations [15,17]. In fact, electrocardiographic findings in LV rupture patients may be related to its type and severity. Electromechanical dissociation (with a diagnostic accuracy that reaches 97%) and bradycardia are features of the acute variety, while new ST-elevation in the affected leads or persistent non-inversion of T-waves may suggest the less noisy "stuttering" type of rupture [9,14]. In this setting, interventions that can dramatically change prognosis can be employed, as long as an accurate diagnosis can be timely established. Clinical course depends mainly on the rate of bleeding and the compliance of the pericardial sac. Signs of restricted ventricular filling may be absent if the effusion develops slowly enough and bleeding ultimately subsides [15,27,30,31].

In a scenario of relatively low threshold for clinical suspicion, the unexpected ECG changes were the key to the diagnosis. Otherwise, patient was only to be exposed to the natural history of the disease which, at this point, may be rather unpredictable [15-17]. In our institution, the risk stratification protocol after an acute MI includes an echocardiographic examination. However, unless a complicated clinical course ensues, it is performed only after the patient has stepped down to the ward. Thus, no additional investigations were to be made at that time.

In those who are haemodynamically unstable, which was not the case of our patient, right heart catheterization will usually provide useful information and may help in the differential diagnosis with other life threatening complications, such as VSD. In the typical patient, right atrial pressure will be comparable to pulmonary capillary wedge pressure [17].

Emergency pericardiocentesis has the potential for the identification of haemopericardium and relief of cardiac tamponade. Despite rarely seen in the acute MI setting, cardiac tamponade may be caused by serous or serohaemorrhagic pericardial effusions; also, the risk of chamber puncture and the theoretical possibility of "thrombus displacement" and "decompression" of the contained rupture, can, not only hamper the diagnostic accuracy of pericardiocentesis, but also make it potentially harmful. Given these considerations, we strongly feel that this procedure should not be "routinely" used as a diagnostic tool and should be kept for situations of absolute need for tamponade relief [15].

If a mechanical complication is suspected, emergency bedside transthoracic and/or contrasted transesophageal echocardiograms are considered the gold standard diagnostic tools [32].

Pericardial effusion is the most common finding, usually with echodense intrapericardial echoes. The absence of pericardial effusion in a patient with AMI excludes the diagnosis of myocardial rupture, but its presence as an isolated finding in this context does not definitively confirm the diagnosis. Right atrial and right ventricular wall compression as well as Doppler signs of a compromised ventricular filling, can also be identified by 2D echocardiography. Direct signs of rupture like a myocardial tear, as in this case, will be seen only occasionally [15,17]. In a prospective study involving 1247 MI patients (33 with subacute LV rupture), the presence of cardiac tamponade, pericardial effusion greater than 5 mm, high density intrapericardial echoes or right atrial or right ventricular wall compression had a high diagnostic sensitivity (≥ 70%) and specificity (≥ 90%). Despite data reporting on high positive predictive values of intrapericardial echoes and echocardiographic signs of tamponade alone, still the number of false-positive findings for every single variable may be relatively high (>20%) [15,30]. However, when combined with syncope, in the appropriate clinical setting, their diagnostic accuracy may reach 100% [15].

In our case, Doppler examination suggested an abnormal intramyocardial tract, but could not definitely demonstrate active bleeding into the pericardium. The detection of blood flow at the site of rupture may not be feasible in many cases, as the myocardial tear is most often anfractuous and sometimes covered by thrombi and thus blood flow velocities may be low. Despite the potential ability of contrast echocardiography to decrease the number of false-positive diagnoses [33-35] – by showing leakage of contrast from the left ventricle into the pericardial effusion – it was of no help in our patient.

The role of new imaging techniques in this setting, such as cardiac MRI, although promising, remains observational and anecdotic [36-38]. Trials assessing the relative value of echocardiography and high resolution CT scans or cardiac MRI are limited not only by clinical, but also by obvious ethical and logistic issues. The wide availability of ultrasound makes this technique "difficult to beat" in the clinical ground, as the expensive technology and expertise needed for cardiac MDCT or MRI imaging are not promptly available in the majority of the hospitals, even in those where cardiac surgery is promptly available. Nevertheless, we should emphasize the high grade of concordance between echocardiographic and MRI images (Figure 4 and Figure 5). Although MRI had, in our case, a paramount importance in the establishment of the diagnosis and in the decision to proceed to surgical treatment, standard and/or contrast enhanced echocardiograms, in expert hands, will generally be trusted as the ultimate diagnostic tools.

### Management and outcome

Despite cases of a fairly good long term survival following a conservative approach have been described [16,17], there is general agreement in that surgery provides the only potentially definitive treatment option and that the diagnosis of subacute LVFWR usually requires a surgical decision [17]. However, the finding that patients presenting with LV pseudoaneurisms might have survived to subacute forms of LV rupture (with only mild hypotension or asymptomatic pericardial effusions) may argue against the absolute need for surgery, suggesting that a conservative management can be an acceptable choice in carefully selected patients, namely those at high surgical risk.

One prospective study reported a 44% survival rate among MI patients with confirmed or strongly suspected LVFWR who survived resuscitation maneuvers after electromechanical dissociation (EMD) or presented only with hypotension without EMD [17] and were treated conservatively. However, in this study there wasn't a surgically treated group suitable for comparison and the possibility of other causes of cardiac tamponade could not be definitely ruled out. Medical management included pericardiocentesis as needed, prolonged bed rest and strict control of LV pressure (preferably with beta blockers) and avoidance of obstipation. Blinc *et al*., in a retrospective analysis, reported much poorer results with a survival rate of only 10% [16]. Intra-aortic balloon pumping has also been employed effectively in unstable patients unresponsive to inotropic agents and fluid replacement [31] but some have found its use to be debatable, unless persistent ischemia and/or LV pump failure also ensues [17,15].

The very high surgical risk, which can be unacceptable if the procedure is to end up as "blank" sternotomy, is the major drawback posed to surgeons. Thus, all efforts should be made to confirm the diagnosis in order to reduce the number of false positives. The goals of surgery are to stop bleeding, to relieve cardiac tamponade and to prevent a second rupture. When possible, off-pump sutureless techniques using biocompatible glues and patches to cover the necrotic/ruptured area are increasingly being preferred to infarctectomy and direct myocardial suture, and are thought to yield better results [14,15,39,40]. However, some reports have suggested that inferior-lateral ruptures – as in the case of our patient – may be adequately treated with direct suture. Coronary artery bypass grafting is also indicated as needed. Emergency coronary angiography in order to determine which coronary arteries to bypass is warranted in the subacute setting, although the possibility of proceeding directly to surgery and perform empirically based bypassing of all of the major epicardial coronary arteries as also been described [41].

Despite high perioperative mortality rates (33 to 55%), a rather well preserved post-procedure left ventricular function, as well as a fairly good functional long term prognosis are increasingly being reported [15,39-41].

## Conclusion

This case illustrates that LVFWR is not always fatal especially in patients with the subacute form of presentation, which occurs in about one third of the cases. The diagnosis can be made accurately using bedside echocardiography, as long as clinicians are aware of the risk factors, clinical signs and other subtle manifestations, such as ECG changes. Because the clinical course is unpredictable, excessive confidence in the stability of the patient can be misleading and even fatal. Considering that surgery yields fairly good immediate and long term results, we feel that surgical treatment should be promptly advised to most patients regardless of the clinical status, unless the diagnosis is doubtful and the surgical risk is unacceptably high.

## List of Abbreviations

PCI – Percutaneous Intervention

CRP – C-Reactive Protein

MRI – Magnetic Resonance Imaging

NSAIDs – Non-Steroid Anti-Inflammatory Drugs

## Competing interests

The author(s) declare that they have no competing interests.

## Authors' contributions

LR was responsible for the initial clinical assessment of the patient and diagnostic workup, performed the literature review and wrote the manuscript. MJA performed the bedside echocardiogram, participated in the drafting, and revised the manuscript for important intellectual content. JF and CA participated in the clinical assessment of the patient and diagnostic workup, and revised the manuscript for important intellectual content. RC revised the document for important intellectual content. MC performed the transesophageal echocardiogram. NJT performed the cardiac MRI. MA performed the surgical procedure. JAS gave final approval to the manuscript. All authors read and approved the final manuscript.

## Supplementary Material

Additional file 1 **Movie 1 – **2D-Echo: Subcostal view showing echodense pericardial effusion. Echoes correspond to blood cloths and/or fibrin. Click here for file

Additional file 2 **Movie 2 – **2D-Echo: Parasternal short axis view showing a myocardial tear in the left ventricular inferior wall. Click here for file

Additional file 3 **Movie 3 – **2D-Echo: Two-chamber view showing the myocardial tear in the left ventricular inferior wall. Note that it is particularly evident during diastole. Click here for file

Additional file 4 **Movie 4 – **2D-Echo: Magnified two-chamber view showing the myocardial tear in the left ventricular inferior wall. Note that it is particularly evident during diastole. Click here for file

Additional file 5 **Movie 5 – **2D-Echo: Apical two-chamber view with Power-Doppler suggesting blood flow across the inferior myocardial wall. Click here for file
